# Rheumatoid Arthritis Disease Activity Is Determinant for ABCB1 and ABCG2 Drug-Efflux Transporters Function

**DOI:** 10.1371/journal.pone.0159556

**Published:** 2016-07-21

**Authors:** Yemil Atisha-Fregoso, Guadalupe Lima, Virginia Pascual-Ramos, Miguel Baños-Peláez, Hilda Fragoso-Loyo, Juan Jakez-Ocampo, Irazú Contreras-Yáñez, Luis Llorente

**Affiliations:** 1 Division of Medicine, Instituto Nacional de Ciencias Médicas y Nutrición Salvador Zubirán, México City, México; 2 Department of Immunology and Rheumatology, Instituto Nacional de Ciencias Médicas y Nutrición Salvador Zubirán, México City, México; University of Birmingham, UNITED KINGDOM

## Abstract

**Objective:**

To compare drug efflux function of ABCB1 and ABCG2 transporters in rheumatoid arthritis (RA) patients with active disease and in remission.

**Methods:**

Twenty two active RA patients (DAS28 ≥3.2) and 22 patients in remission (DAS28<2.6) were selected from an early RA clinic. All patients were evaluated at study inclusion and six months later. ABCB1 and ABCG2 functional activity was measured in peripheral lymphocytes by flow cytometry. The percentage of cells able to extrude substrates for ABCB1 and ABCG2 was recorded.

**Results:**

Active patients had higher ABCB1 and ABCG2 activity compared with patients in remission (median [interquartile range]): 3.9% (1.4–22.2) vs (1.3% (0.6–3.2), p = 0.003 and 3.9% (1.1–13.3) vs 0.9% (0.5–1.9) p = 0.006 respectively. Both transporters correlated with disease activity assessed by DAS28, rho = 0.45, p = 0.002 and rho = 0.47, p = 0.001 respectively. Correlation was observed between the time from the beginning of treatment and transporter activity: rho = 0.34, p = 0.025 for ABCB1 and rho = 0.35, p = 0.018 for ABCG2. The linear regression model showed that DAS28 and the time from the onset of treatment are predictors of ABCB1 and ABCG2 functional activity, even after adjustment for treatment. After six months we calculated the correlation between change in DAS28 and change in the functional activity in both transporters and found a moderate and significant correlation for ABCG2 (rho = 0.28, p = 0.04) and a non-significant correlation for ABCB1 (rho = 0.22, p = 0.11).

**Conclusions:**

Patients with active RA have an increased function of ABCB1 and ABCG2, and disease activity is the main determinant of this phenomena.

## Introduction

Rheumatoid arthritis (RA) is a chronic, systemic, inflammatory joint disease that leads to bone and cartilage destruction, as well as to a wide variety of extra articular manifestations [1]. The impact of RA is considerable, as it affects young adults who may suffer work disability as a consequence [2,3].

Early, aggressive treatment, aimed at reaching remission with disease-modifying antirheumatic drugs (DMARD) has a favorable impact on patient outcomes [4–6]. More than 50% of patients, however, do not respond adequately to the conventional initial treatment; this lack of response is associated with the use of potentially more toxic combinations, greater costs and worse clinical outcomes [7].

One of the mechanisms that have been proposed as a potential cause of resistance or failure to treatment in RA is the resistance to drugs mediated by transporters that decreases intracellular drug concentration by effluxing them from the intracellular space. The first of these transporters to be described was the permeability glycoprotein (P-gp), also known as ABCB1, the product of the MDR-1 gene, which belongs to the superfamily of ABC (ATP-binding cassette) transporters [8,9]. Another transporter member of this family which has also been studied is the ABCG2 or Breast Cancer Resistant Protein (BCRP1) [10]. The specificity of both transporters is variable, and has been physiologically related to hormone secretion and expulsion of bacterial toxins from cells; the increase of function of both transporters also has as a consequence the extrusion of many drugs from the intracellular space.

These transporters have been studied mainly in cancer, even though the acquired knowledge related to their role in the response to oncological therapy has expanded to antiviral [11] and immunosuppressive [12] drugs. The main importance of these transporters in RA is that its known substrates include, for ABCG2, methotrexate, leflunomide and sulfasalazine, and for ABCB1, prednisolone and chloroquine [13], all these drugs being a fundamental part of the treatment of RA. Within the clinical context of RA there have been few studies analysing the determinants of functional activity of these transporters in patients, and most of them have been carried out on ABCB1.

The aim of the present study was to search for the association between disease activity and treatment (corticosteroids and/or DMARD) and with the functional activity of the ABCB1 and ABCG2 transporters.

## Materials and Methods

### Study design and variables

This is a case and control study nested in a cohort from the early RA clinic at a tertiary care centre in Mexico City. The clinic includes patients with symptoms of less than 12 months old who continue with an indefinite, structured follow [14].

For this study, we consecutively included 22 cases and 22 controls obtained from the early RA clinic. All of these patients had a confirmed RA diagnosis according to the ACR/EULAR classification criteria [15,16]. A case was considered to be any patient having a disease activity index (DAS28) > 3.2 at the time of inclusion in the study [17], receiving therapeutic and stable (at least two months) doses of DMARD (active RA). A control was considered to be a patient with RA, also receiving stable treatment and in remission, i.e., DAS28<2.6.

A second control group was also included. It consists of 8 patients with RA, who had started treatment for RA less than two weeks before their inclusion in the study. Remarkably 4 of them had not begun treatment at the time of the basal sample (recent diagnosis).

For determination of the normal values of ABCB1 and ABCG2 30 healthy volunteers were also studied, 27 women and three men (age range 19–38 years, mean 26.8 years).

This project was approved by the Instituto Nacional de Ciencias Médicas y Nutrición ethics committee and all subjects were informed about the objectives of the study and gave their written consent to participate.

### Patient assessment and obtaining of samples

At the time of inclusion in this study and six months later, we registered sociodemographic data, presence of comorbidities, disease activity, treatment and adherence to it. Disease activity was prospectively determined by the identification and count of painful and swollen joints [17], and by the determination of acute phase reactants (erythrocyte sedimentation rate [ESR] and C reactive protein [CRP]). These data were used to calculate the DAS28-VSG index. Depending on DAS28, patients were classified as in remission (<2.6), with low disease activity (2.6–3.2) or with active disease (>3.2).

For each patient the drugs doses at the inclusion in this study were determined, as was also the accumulated dose during the previous year. Analysed drugs were: corticosteroids (equivalent doses of prednisone), methotrexate, chloroquine (or hydroxichloroquine), sulfasalazine and leflunomide.

For the measurement of the functional activity of ABCB1 and ABCG2 by flow cytometry a 5 mL peripheral blood sample was taken at baseline and six months later.

For flow cytometric analysis of ABCB1 and ABCG2 function, peripheral blood mononuclear cells were isolated by gradient centrifugation on Lymphoprep (Oslo, Norway) from 5 mL EDTA-anticoagulated whole blood. After two washes, cells were adjusted at a concentration of 3 × 10^6^ cells/ mL in phosphate-saline. For the measurement of ABCB1 activity, 60 μL of 400 μM daunorubicin (DNR) (Sigma-Aldrich, San Louis, MO) -a fluorescent substrate of ABCB1- were loaded to the cells. Samples were divided into three aliquots of 1 x 10^6^ cells/mL; one of them was incubated for 1 hour minutes in crushed ice for baseline DNR uptake; another one incubated at 37°C for 1 hour in a water bath in order to allow DNR-efflux, and the last one incubated in the presence of 10 μL of 5 mM verapamil (Sigma-Aldrich) -a specific inhibitor of ABCB1- at 37°C for 1 hour in a water bath to verify the specificity of DNR extrusion.

**Fig 1:**
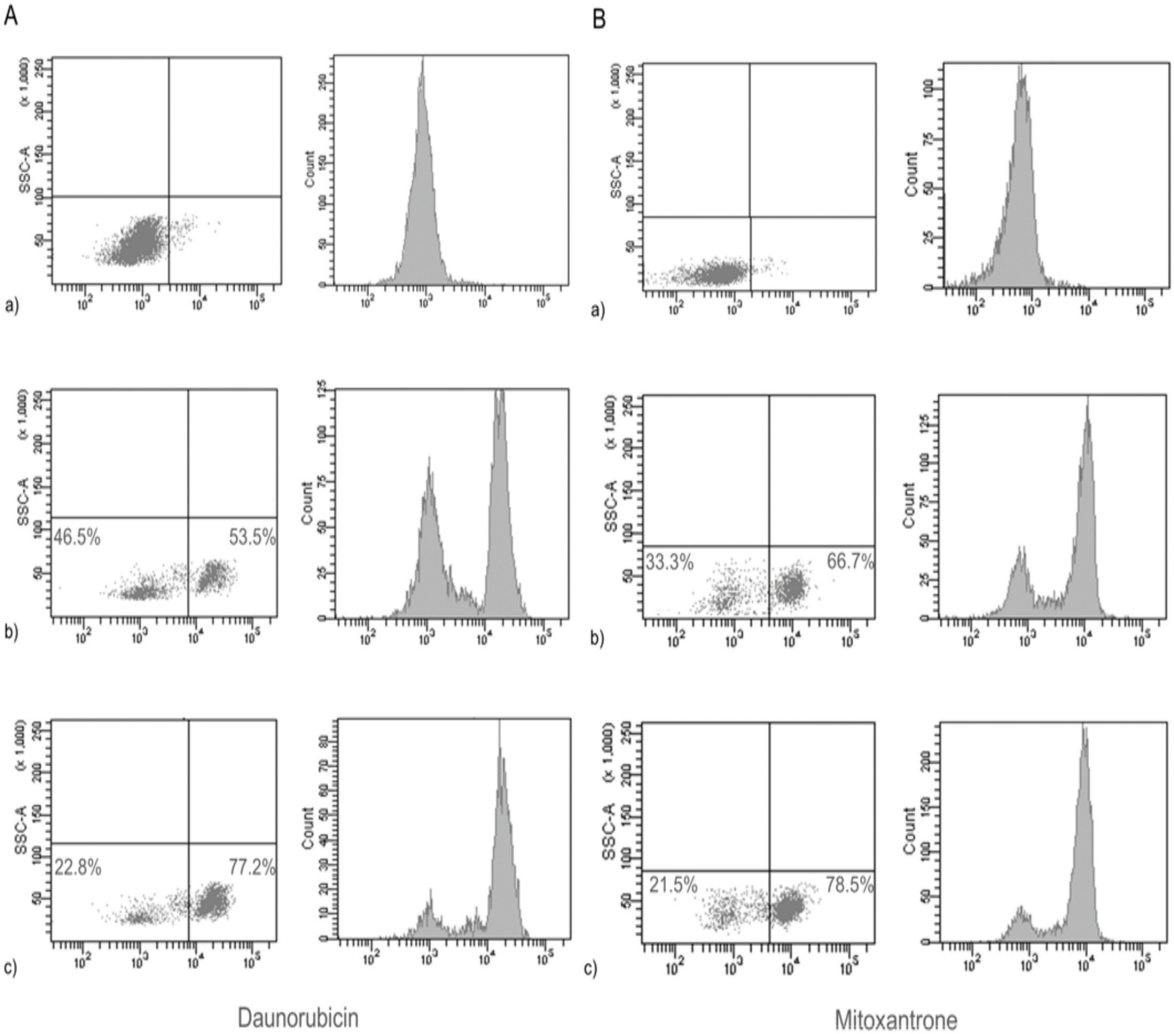
Representative flow cytometric analyses of daunorubicin and mitoxantrone extruding lymphocytes. Panel A. Representative flow cytometric analyses of DNR extruding lymphocytes. The figure displays representative cytograms and their corresponding histograms obtained by cells incubated at 4°C (a) (negative control), 37°C (b) and 37°C (c) in the presence of verapamil. The non DNR-effluxing (fluorescent cluster at the right) and the DNR-effluxing (fluorescent cluster at the left) lymphocytes are clearly evident in either cytograms or histograms. (b): active RA patient with 46.5% lymphocytes able to extrude DNR; (c) Inhibitory effect of verapamil with only 22% of effluxing cells. Panel B. Representative flow cytometric analyses of mitoxantrone-extruding lymphocytes. The figure displays representative cytograms and their corresponding histograms obtained by cells incubated at 4°C (a) (negative control), 37°C (b) and 37°C (c) in the presence of KO143. The non-mitoxantrone effluxing-(fluorescence cluster at the right) and the mitoxantrone-effluxing (fluorescence cluster at the left) lymphocytes are clearly evident in either cytograms or histograms. b): active RA patient with 33.3% lymphocytes able to extrude mitoxantrone; (c) Inhibitory effect of KO143 with only 21.5% effluxing lymphocytes.

For the measurement of ABCG2 activity 20 μL of 500 μM mitoxantrone (Sigma-Aldrich)–a fluorescent substrate of ABCG2- were loaded to 3 x 10^6^ cells/mL. Samples were divided into three aliquots of 1 x 10^6^ cells/mL; one of them was incubated for 1 hour in crushed ice for baseline mitoxantrone uptake; another one incubated at 37°C for 1 hour in a water bath in order to allow mitoxantrone efflux, and the last one incubated in the presence of 100 μL of 10 μM KO143 (Sigma-Aldrich)–a specific inhibitor of ABCG2- at 37°C for 1 hour in a water bath to verify the specificity of mitoxantrone extrusion. Analysis were performed immediately on a FACS Canto II (BD Biosciences, San Jose, CA) using the BD FACS Diva software (Fig 1).

A lymphocyte cluster was gated according to side and forward scatter characteristics. To avoid differences in size or granularity among lymphocyte subpopulations, a narrow gate was set in the middle of the first one. Event count was stopped when 20 000 events in this latter gate were achieved. Results were expressed as the percentage of DNR or mitoxantrone-effluxing lymphocytes (low fluorescent cells). Overfunction of ABCB1 and ABCG2 was defined as a percentage greater than the mean value from the healthy control group plus two standard deviations (i.e. 4.22% and 4.09%, respectively). Inhibition by verapamil and KO143 ranged from 43% to 85% and 38% to 87% respectively.

### Statistical analysis

For comparison between groups, we used either Student’s T test, Mann-Whitney U test or Wilcoxon signed rank test, as appropriate. The correlation between disease activity and treatment (doses at inclusion or accumulated during the previous year) with the measurement of transporters activity was defined with Spearman’s rho test. For multiple group comparison we used one-way ANOVA or Kruskal Wallis test. Significance was set at a two-tailed value of p< 0.05.

A multivariate analysis with a linear regression model was performed to establish the independent association of disease activity with the value of both transporters activity. For this adjustment, cumulative doses of glucocorticoids and DMARD and duration of treatment were included in the model. For calculation of coefficient of determination we constructed a model with variables with p ≤ 0.10 in the univariatecorrelation analysis. Results are reported as β coefficient (confidence interval 95%) and p value, the coefficient of determination (R^2^) for the model is reported as well. The variance inflation factor was used to rule out collinearity.

The statistical program SPSS version 20.0 was used (SPSS, Chicago, IL, USA).

## Results

### Study population

Table 1 shows the most relevant characteristics of the population at the time of inclusion in our study. As expected, when compared with controls, cases had a greater clinical disease activity, with a DAS28 (mean ± SD) at inclusion of 4.8 ± 1.2 vs. 1.1 ± 0.5 (p<0.001) and higher serological activity with CRP concentration (mean ± SD) of 1.2 ± 1.2 vs. 0.4 ± 0.5 mg/dL (p = 0.018).

**Table 1:** Clinical and Demographic Characteristics.

	Active RA (n = 22)	Remission RA (n = 22)	Recent diagnosis RA (n = 8)	P Value
Age (years)	46.1 ± 12.3	36.7 ± 10.4	43.7 ± 10.8	P = 0.01* P = 0.027†
Female n (%)	21 (95%)	20 (91%)	8 (100%)	P = 0.6†
Time since disease onset	5 ± 4.2 years	4.5 ± 3.8 years	122 ± 56 days	P = 0.77*
Time since treatment onset	4 ± 3.4 years	4 ± 3.7 years	5.9 ± 8.1 days	P = 0.97*
CRP	1.2 ± 1.2	0.4 ± 0.5	0.7 ± 0.8	P = 0.017†
Smoking n (%)	2 (9%)	2 (9%)	1 (12%)	P = 0.96†
DAS28 basal	4.8 ± 1.2	1.1 ± 0.5	5.4 ± 0.9	P<0.001* P<0.001‡
**TREATMENT**				
Medications use at inclusion				
PDN	17 (77%)	17 (77%)	5 (62%)	P = 1*
Methotrexate	17 (77.3%)	21 (95.5%)	7 (87.5%)	P = 0.18*
Sulfasalazine	6 (27.3%)	6 (27.3%)	1 (12.5%)	P = 1*
Hydroxychloroquine	8 (36.4%)	8 (36.4%)	4 (50.0%)	P = 1*
Drugs doses at inclusion				
PDN	6.8 ± 5.8	4.8 ± 3.4		P = 0.4*
Methotrexate	17 ± 10.5	21.5 ± 5.7		P = 0.42*
Sulfasalazine	727.3 ± 1241.4	568.2 ± 979.5		P = 0.76*
Hydroxychloroquine	59.1 ± 81.1	52.3 ± 79.4		P = 0.78*
Cumulated doses during previous year				
PDN	2321 ± 2169.4	1638 ± 1181		P = 0.310*
Methotrexate	822 ± 564	1055 ± 337		P = 0.211*
Sulfasalazine	180 ± 319	217 ± 398		P = 0.978*
Hydroxychloroquine	17016 ± 24557	21650 ± 27523		P = 0.758*

Values represent mean ± standard deviation or n (%).

RA: Rheumatoid arthritis, CRP: C-reactive protein, PDN: Prednisone. Active RA: Patients with DAS 28 ≥ 3.2, remission: DAS 28 ≤ 2.6. Recent diagnosis RA: patients with less than two weeks with DMARD or glucocorticoids.

* Active vs remission RA.

^†^ Three groups comparison.

^‡^ Remission vs recent diagnosis RA.

There were no differences between groups in the glucocorticoids or DMARD doses at the inclusion in this study, or in the accumulated drug doses during the year prior to the study (Table 1).

### Transporters activity at the onset of the study

**Fig 2:**
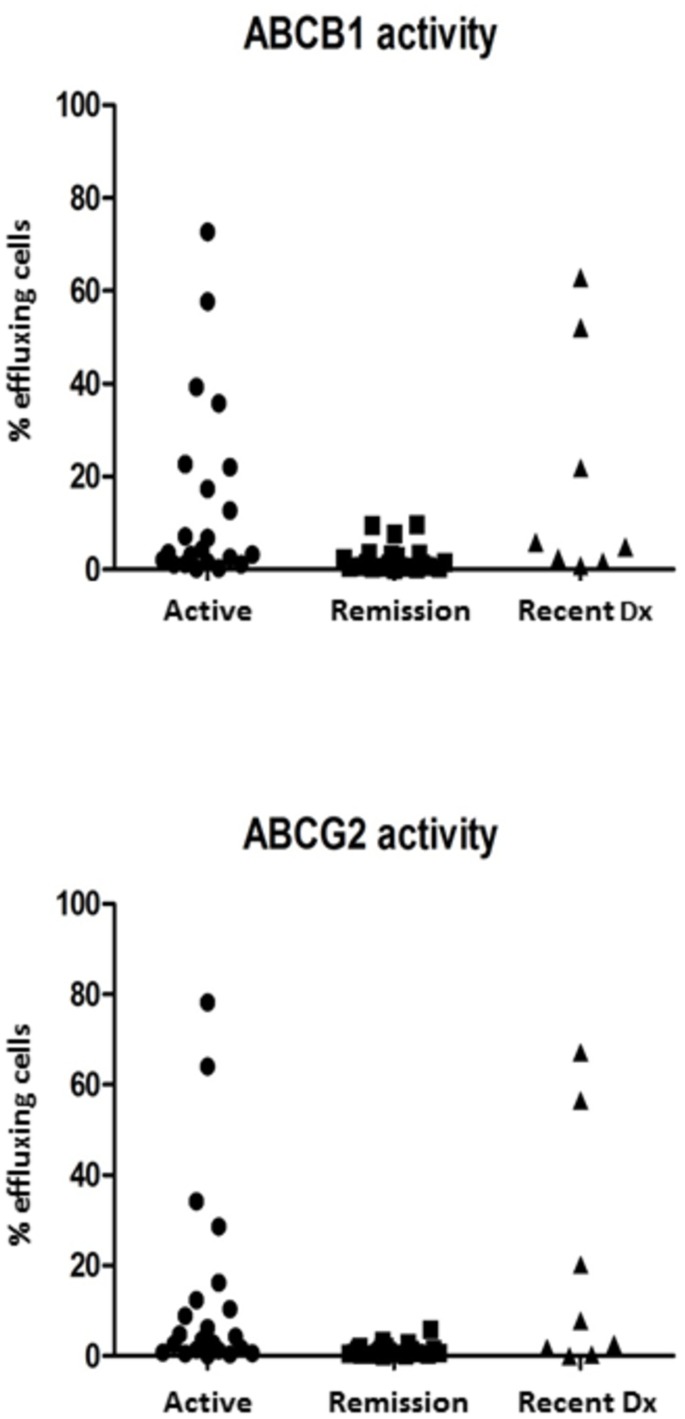
ABCB1 and ABCG2 transporters activity in active RA patients, in remission, and in those with recent diagnosis. Shown are the percent effluxing lymphocytes from all patients studied in each group.

The functional activity expressed as percentage for each transporter (median [interquartile range]) was greater in patients with DAS28 > 3.2 than in patients in remission, for ABCB1 3.9% (1.4–22.2) vs (1.3% (0.6–3.2), p = 0.003 and 3.9% (1.1–13.3) vs 0.9% (0.5–1.9) p = 0.006 (Fig 2).

Function in both transporters correlated significantly with disease activity assessed by DAS28: for ABCB1 rho = 0.45, p = 0.002 and ABCG2 rho = 0.47, p = 0.001. There was no correlation between the functional activity of each transporter and the cumulated drug doses or the doses at sampling of the various drugs. A correlation was observed, however, between the time from the beginning of treatment and transporter activity: rho = 0.34, p = 0.025 for ABCB1 and rho = 0.35, p = 0.018 for ABCG2.

### Linear regression model

The linear regression model with the basal data of all 44 patients showed that DAS28 and the time from the beginning of treatment are functional activity predictors for ABCB1 and ABCG2. This association remains significant even after adjusting for treatment (doses at inclusion or accumulated doses of the administered drugs). Table 2 shows the results of the model including the significant variables i.e. DAS28 and time from the onset of treatment.

**Table 2:** Linear regression model.

Transporter	Variables	β	IC 95%	p	R^2^
ABCB1	Basal DAS28	3.380	1.426–5.335	0.001	0.30
	Time since treatment onset (years)	1.438	0.282–2.595	0.016	
ABCG2	Basal DAS28	3.324	1.278–5.371	0.002	0.27
	Time since treatment onset (years)	1.363	0.152–2.574	0.028	

#### Recently diagnosed patients

With the aim of establishing whether RA patients with a recent onset to their treatment–and thus with minimal exposure to drugs—have an increased transporter activity, we included 8 patients with fewer than two weeks from admission into the RA clinic. In four of them, the sample was obtained before the onset of any treatment whatsoever, and in the remaining four within two weeks of the beginning of treatment. In these patients the DAS28 activity index was 5.4 ± 0.9. The median activity of ABCB1 was 5.4 IQR (1.8–44.5) (p = 0.012 vs inactive) and of ABCG2 5.2 (0.7–47.4) (p = 0.037 vs inactive). (Fig 2).

#### Follow up after six months

After six months of follow up, out of the 22 patients that were initially active, 10 remained active, 2 were found to have low activity and 10 were in remission. Out of the 22 patients that were initially inactive, after six months 18 remained in remission, 3 had low activity and 1 became active.

In 15 patients there was a decrease in DAS28 after 6 months. Interestingly, no one of them had an increase in ABCG2 activity (basal vs 6 months), and only one of these patients showed an increase in ABCB1 activity (basal vs 6 months).

We calculated the correlation between change in DAS28 (difference between basal DAS28 minus DAS28 after 6 months) and change in the functional activity in both transporters (differences between basal value minus value after 6 months). We found a moderate and significant correlation for ABCG2 (rho = 0.28, p = 0.04) and a non-significant correlation for ABCB1 (rho = 0.22, p = 0.11).

Taking into account only the 22 patients that had active disease at the onset of the study, those with greater basal values of ABCB1 or ABCG2 (i.e., those showing values above the median), had no statistically significant difference in DAS28 after 6 months, when compared with those patients with values below the median: 3.5 (IQR 2.2–4.1) vs 2.6 (IQR 1.8–4.4); p = 0.43 for ABCB1 and 3.5 (1.9–4.1) vs 2.6 (2–4.7); p = 0.89 for ABCG2.

## Discussion

This is the first study analysing longitudinally the activity of the transporters ABCB1 and ABCG2 in peripheral lymphocytes of RA patients. These transporters, by including amongst their substrate several of the most important drugs used in treating RA, may have some bearing on the refractoriness to treatment observed in some patients.

Our results show an increased functional activity of both transporters in peripheral lymphocytes of patients with active RA, and a correlation between both values. These results are in agreement with previous reports which had found evidence of an increased activity of ABCB1 in peripheral lymphocytes from patients with active RA [18,19]. Regarding ABCG2, there have only been reports of an increase in the expression of ABCB1 and ABCG2 in macrophages in synovial tissue in patients with RA [20], but this has not been studied in peripheral mononuclear cells.

We also found a correlation between the transporters’ activity and the time elapsed from the beginning of treatment, which tallies with reports which had previously found an association between higher expression of ABCB1 with prednisolone treatment [21], and an increase in ABCB1 expression in lymphocytes from synovial tissue from patients previously treated with DMARD [22].

This association of ABCB1 and ABCG2 function with disease activity and treatment accepts several explanations. It is important to establish whether in RA patients the greater activity of transporters is directly associated with disease activity or whether it is secondary to a greater exposure to drugs, since it is considered that these transporters are inducible [23,24] and patients with a more active disease receive more treatment.

Our results suggest a mixed behaviour: in the multivariate analysis the main determinant of ABCB1 and ABCG2 function was disease activity, and this association remains significant after adjustment for treatment. Even though we could not find a direct association with any of the drugs used in the treatment of RA, there was a correlation of transporters’ activity with the time from the onset of treatment (independent of RA duration). This association can be ascribed to a complex interaction amongst all the drugs received, which over time induces a greater activity in transporters. These two variables explain about 30% of the variability of the transporters’ activity.

Additionally, recently diagnosed RA patients, despite not having received treatment had greater transporter activity than inactive patients with greater drug exposure. This finding had already been reported for ABCB1 [19], although it had not been previously reported for ABCG2. This fact strengthens the role of disease activity as a determinant factor in the transporters’ function in some patients.

This is an interesting phenomenon, and even though it could be due to an intrinsic characteristic of lymphocytes from RA patients, it is worth pointing out that one of the physiological functions of both ABCB1 and ABCG2 is cellular detoxification, including the active extrusion of inflammatory mediators such as TNF-α, IL-2, IL-12 and IFN-γ [24], and it is possible that the chronic exposure to increased levels of them could generate a greater transporter activity.

It has been suggested that as the disease activity decreases, so does that of the transporters [25,26]. Notwithstanding this, we only found a weak correlation between the change in DAS28 at 6 months and the change in the value of the transporters activity, which was only significant for ABCG2. Nevertheless, six months later there was still a significant association between transporter function and disease activity. This could be explained by a delayed response of the transporters function to a decrease in disease activity, but studies with a longer follow up are needed to clarify this point.

One of the main points of interest concerning the increase in the activity of these transporters is whether it is associated with a phenotype of clinical resistance to treatment since, for example, ABCG2 has been associated *in vitro* to resistance to sulfasalazine, lefluonomide and methotrexate [27,28]. We did not find any difference between DAS28 at six months in patients with active disease and with a greater transporter activity when compared with those with active RA and a lesser transporter activity. It is worth noting, however, that this study was not empowered to analyse this point. Studies specifically designed to establish whether patients with greater transporter activity have less probabilities of reaching a state of remission of RA when receiving treatment with DMARD are needed.

Interestingly, it has been shown *in vitro* that the blockage of both transporters with chemosensitizers such as verapamil and KO-143 is able to inhibit the function of the transporters and the cellular resistance to some DMARD [29]. Even though there are some inhibitors which are able to revert *in vivo* the resistance phenotype in humans [30], these have been employed in cancer with poor results. There are not sufficient studies in patients with autoimmune diseases, where due to a different background of cell alterations, we could expect different results and argue for their beneficial therapeutic potential (as long as they are used in addition to the employed regimes), as optimizers of the pharmacological action of DMARD.

Our study has some limitations, such as having studied peripheral lymphocytes as a whole, which did not allow us to establish whether a specific population is responsible for the resistance phenotype, nor could we assess the functional alterations that may accompany greater activity by ABCB1 and/or ABCG2.

It is interesting, however, that these changes are to be found in lymphocytes from peripheral blood and not exclusively located in the synovia, being that, although peripheral blood lymphocytes from patients with RA do not coincide within a general activation state, there have been reports of alterations in their proliferation and differentiation rate, with a decrease of naïve CD4 cells [31], and certain memory T-lymphocytes populations show an increase in the expression of ABCB1, whose functional meaning has not yet been ascertained [32].

Further, we had a relatively small number of patients, and while we were able to establish differences in ABCB1 and ABCG2 between patients with active and inactive disease, we could not solve other questions, such as the prognostic value of different levels of transporter activity.

On the other hand, the current study has several strengths: the population under study contains representative groups of the disease, and patients were part of a prospective cohort with a rigorous follow up, which enabled us to investigate the possible association of treatment and activity with the transporters functional activity.

Another point worth remarking upon is the process employed in determining the transporters activity. The only validated method to measure directly the transporters function in cells in suspension is the extrusion of fluorescent compounds dependent on these molecules, e.g., daunorubicin, rhodamine 123, mitoxantrone. With these substances it is possible to measure the individual capacity of each cell to expel drugs substrates of ABCB1 and ABCG2 [33], and combined with the inhibition analysis it is possible to attribute the extrusion to a specific transporter. Clinically it is more important to establish the function of the transporters than just their presence in the membrane or the expression of the gene, which could not be representative of the biological phenomena of extrusion of relevant compounds. Also, measuring the transporters activity using peripheral blood facilitates the obtaining of multiple samples.

Summing up, our results demonstrate that patients with active rheumatoid arthritis have an increased function of ABCB1 and ABCG2, and that disease activity is the variable with the strongest association with this phenomena, even though there is also an association with treatment duration. Certainly, more studies are needed to identify the prognostic value of this increment in transporters function, and to establish the causal and temporal association between both phenomena, as there could exist a circular relationship, where a greater activity from the transporters’ conditions a greater disease activity, and a greater disease activity induces a greater transporters’ activity. These items deserves to be explored in depth.

## References

[1] Fleming A, Benn RT, Corbert M, Wood PH. Early rheumatoid disease. II. Patterns of joint involvement. Ann Rheum Dis 1976;35: 361–4. 970995 10.1136/ard.35.4.361 PMC1007397

[2] Cooper NJ. Economic burden of rheumatoid arthritis: A systematic review. Rheumatology 2000;39: 28–33. 10662870 10.1093/rheumatology/39.1.28

[3] Young A, Dixey J, Kulinskaya E, Cox N, Davies P, Devlin J, et al Which patients with early arthritis stop working? Results of five years' follow up in 732 patients from the Early RA Study (ERAS). Ann Rheum Dis 2002; 61: 335–40. 11874837 10.1136/ard.61.4.335 PMC1754067

[4] Emery P, Salmon M. Early rheumatoid arthritis: to aim for remission? Ann Rheum Dis 1995; 54: 944–7. 8546524 10.1136/ard.54.12.944 PMC1010056

[5] Paulus HE. Defining remission in rheumatoid arthritis: what is it? Does it matter? J Rheumatol 2004;31: 1–4. 14705208

[6] Huizinga T, Knevel R. Rheumatoid arthritis: 2014 treat-to-target RA recommendations-strategy is key. Nat Rev Rheumatol 2015;11: 509–11. 10.1038/nrrheum.2015.98 26195337

[7] Haraoui B. The anti-tumor necrosis factor agents are a major advance in the treatment of rheumatoid arthritis. J Rheumatol (Suppl) 2005; 72:46–7. 15660467

[8] Goda K, Bacsó Z, Szabó G. Multidrug resistance through the spectacle of P-glycoprotein. Curr Cancer Drug Targets 2009; 9: 281–9. 19442049 10.2174/156800909788166493

[9] Ueda K, Cornwell MM, Gottesman MM, Pastan I, Roninson IB, Ling V, et al The mdr1 gene, responsible for multidrug-resistance, codes for P-glycoprotein. Biochem Biophys Res Commun 1986;141: 956–62. 2880583 10.1016/s0006-291x(86)80136-x

[10] Litman T, Druley TE, Stein WD, Bates SE. From MDR to MXR: new understanding of multidrug resistance systems, their properties and clinical significance. Cell Mol Life Sci 2001;58: 931–59 11497241 10.1007/PL00000912 PMC11337370

[11] Mohri H, Markowitz M. In vitro characterization of multidrug-resistant HIV-1 isolates from a recently infected patient associated with dual tropism and rapid disease progression. J Acquir Immune Defic Syndr 2008;48: 511–21. 10.1097/QAI.0b013e31817ecb31 18645523 PMC3725736

[12] Jansen G, Scheper RJ, Dijkmans BA. Multidrug resistance proteins in rheumatoid arthritis, role in disease-modifying antirheumatic drug efficacy and inflammatory processes: an overview. Scand J Rheumatol 2003;32: 325–36. 15080263 10.1080/03009740310004333

[13] van der Heijden JW, Dijkmans BA, Scheper RJ, Jansen G. Drug Insight: resistance to methotrexate and other disease-modifying antirheumatic drugs-from bench to bedside. Nat Clin Pract Rheumatol 2007;3: 26–34. 17203006 10.1038/ncprheum0380

[14] Pascual-Ramos V, Contreras-Yáñez I, Villa AR, Cabiedes J, Rull-Gabayet M. Medication persistence over 2-years follow-up in a cohort of early rheumatoid arthritis patients: associated factors and relationship with disease activity. Arthritis Res Ther 2009;11: R26 10.1186/ar2620 19228421 PMC2688260

[15] Aletaha D, Neogi T, Silman AJ, Funovits J, Felson DT, Bingham CO 3rd, et al 2010 Rheumatoid arthritis classification criteria: An American College of Rheumatology/European League against rheumatism collaborative initiative. Ann Rheum Dis 2010; 69: 1580–8. 10.1136/ard.2010.138461 20699241

[16] Arnett FC, Edworthy SM, Bloch DA, McShane DJ, Fries JF, Cooper NS, et al The American Rheumatism Association 1987 revised criteria for classification of rheumatoid arthritis. Arthritis Rheum 1988;31: 315–24. 3358796 10.1002/art.1780310302

[17] Prevoo ML. Modified disease activity scores that include twenty-eight-joint counts. Development and validation in a prospective longitudinal study of patients with rheumatoid arthritis. Arthritis Rheum 1995;38: 44–8. 7818570 10.1002/art.1780380107

[18] Llorente L, Richaud-Patin Y, Díaz-Borjón A, Alvarado de la Barrera C, Jakez-Ocampo J, de la Fuente H, et al Multidrug resistance-1 (MDR-1) in rheumatic autoimmune disorders. Part I: Increased P-glycoprotein activity in lymphocytes from rheumatoid arthritis patients might influence disease outcome. Joint Bone Spine 2000;67: 30–9. 10773966

[19] Agarwal V, Mittal SK, Misra R. Expression of multidrug resistance-1 protein correlates with disease activity rather than the refractoriness to methotrexate therapy in rheumatoid arthritis. Clin Rheumatol 2009;28: 427–33. 10.1007/s10067-008-1071-1 19137355

[20] van der Heijden JW, Oerlemans R, Tak PP, Assaraf YG, Kraan MC, Scheffer GL, et al Involvement of breast cancer resistance protein expression on rheumatoid arthritis synovial tissue macrophages in resistance to methotrexate and leflunomide. Arthritis Rheum 2009;60: 669–77 10.1002/art.24354 19248091

[21] Maillefert JF, Maynadie M, Tebib JG, Aho S, Walker P, Chatard C, et al Expression of the multidrug resistance glycoprotein 170 in the peripheral blood lymphocytes of rheumatoid arthritis patients. The percentage of lymphocytes expressing glycoprotein 170 is increased in patients treated with prednisolone. Br J Rheumatol 1996;35: 430–5. 8646432 10.1093/rheumatology/35.5.430

[22] Jorgensen C, Sun R, Rossi JF, Costes J, Richard D, Bologna C, et al Expression of multidrug resistance gene in human rheumatoid synovium. Rheumatol Int 1995;15: 83–6. 7481486 10.1007/BF00262714

[23] Richaud-Patin Y, Soto-Vega E, Jakez-Ocampo J, Llorente L. P glycoprotein in autoimmune diseases. Autoimmun Rev 2004;3: 88–92 10.1016/j.autrev.2003.08.002 15110230

[24] van de Ven R, Oerlemans R, van der Heijden JW, Scheffer GL, de Gruijl TD, Jansen G, et al ABC drug transporters and immunity: novel therapeutic targets in autoimmunity and cancer. J Leukoc Biol 2009;86: 1075–9. 10.1189/jlb.0309147 19745159

[25] Hider SL, Owen A, Hartkoorn R, Khoo S, Back D, Silman AJ, et al Down regulation of multidrug resistance protein-1 expression in patients with early rheumatoid arthritis exposed to methotrexate as a first disease-modifying antirheumatic drug. Ann Rheum Dis 2006;65: 1390–3. 16504991 10.1136/ard.2005.049189 PMC1798314

[26] Tsujimura S, Saito K, Nawata M, Nakayamada S, Tanaka Y. Overcoming drug resistance induced by P-glycoprotein on lymphocytes in patients with refractory rheumatoid arthritis. Ann Rheum Dis 2008;67: 380–8. 17660216 10.1136/ard.2007.070821

[27] van der Heijden J, de Jong MC, Dijkmans BA, Lems WF, Oerlemans R, Kathmann I, et al Acquires resistance of human T cells to sulfasalazine: stability of the resistant phenotype and sensitivity to non-related DMARDs. Ann Rheum Dis 2004;63: 131–7. 14722200 10.1136/ard.2003.006494 PMC1754886

[28] van der Heijden J, de Jong MC, Dijkmans BA, Lems WF, Oerlemans R, Kathmann I, et al Development of sulfasalazine resistance in human T cells induces expression of the myultidrug resistance transporter ABCG2 (BCRP) and augmented production of TNF alpha. Ann Rheum Dis 2004;63: 138–43. 14722201 10.1136/ard.2002.005249 PMC1754889

[29] Márki-Zay J, Tauberné Jakab K, Szerémy P, Krajcsi P. MDR-ABC transporters: biomarkers in rheumatoid arthritis. Clin Exp Rheumatol 2013;31: 779–87. 23711386

[30] Palmeira A, Sousa E, Vasconcelos MH, Pinto MM. Three decades of P-gp inhibitors: skimming through several generations and scaffolds. Curr Med Chem 2012;19: 1946–2025. 22257057 10.2174/092986712800167392

[31] Ponchel F, Morgan AW, Bingham SJ, Quinn M, Buch M, Verburg RJ, et al Dysregulated lymphocyte proliferation and differentiation in patients with rheumatoid arthritis. Blood. 2002;100:4550–6. 12393721 10.1182/blood-2002-03-0671

[32] Butterfield K, Fathman CG, Budd RC. A subset of memory CD4+ helper T lymphocytes identified by expression of Pgp-1. J Exp Med. 1989;169:1461–6. 2564418 10.1084/jem.169.4.1461 PMC2189224

[33] Kapplemayer J, Karazi E, Telek B, Jakab K. “Pros and cons” on how to measure multidrug resistance in leukemias. Leuk Lymphoma 2002;43: 711–17. 12153155 10.1080/10428190290016791

